# Analysis of PANoptosis-Related LncRNA-miRNA-mRNA Network Reveals LncRNA SNHG7 Involved in Chemo-Resistance in Colon Adenocarcinoma

**DOI:** 10.3389/fonc.2022.888105

**Published:** 2022-05-12

**Authors:** Jingjing Huang, Shiyao Jiang, Lu Liang, Hua He, Yueying Liu, Li Cong, Yiqun Jiang

**Affiliations:** ^1^ The Key Laboratory of Model Animal and Stem Cell Biology in Hunan Province, Hunan Normal University, Changsha, China; ^2^ School of Medicine, Hunan Normal University, Changsha, China

**Keywords:** COAD, PANoptosis, lncRNA, metastasis, drug resistance

## Abstract

Colon adenocarcinoma (COAD) is one of the most common malignancies, and its metastatic lesions are the leading cause of death in COAD patients. PANoptosis is a recently identified pathway for programmed cell death implicated in developing COAD. Long non-coding RNAs (lncRNAs) are key regulators of cancer occurrence and progress. Although their function has captured much attention in COAD, the relationship between COAD metastasis-associated lncRNA expression and PANoptosis remains elusive. Therefore, this study aimed to explore the potential regulatory roles of metastasis- and PANoptosis-associated lncRNAs in COAD. Nine lncRNAs associated with metastasis and PANoptosis in COAD were identified from The Cancer Genome Atlas (TCGA) and GEO databases. Their functions were analyzed by multiple bioinformatics methods, and the lncRNA-miRNA-mRNA network was constructed. Multivariate Cox analysis identified one lncRNA (SNHG7) significantly related to COAD prognosis. Subsequent analyses showed its expression correlated with tumor stage and lymph node metastasis. Moreover, drug sensitivity analysis and *in vitro* experiments suggest that lncRNA SNHG7 contributes to drug resistance in COAD. In summary, lncRNA SNHG7 is a potential target for diagnosing and treating COAD and plays a crucial role in regulating apoptosis, metastasis, and drug resistance in COAD.

## Introduction

Colorectal cancer (CRC) is one of the most widespread malignancies, having the third highest incidence and second highest mortality rate among 36 cancers reported by GLOBOCAN 2020 (1). It is particularly common in developed countries, and unfortunately, its incidence is slowly increasing (2). Colon adenocarcinoma (COAD) is the most common subtype of CRC, and its diagnosis and treatment have seen recent rapid development. However, since metastatic cancer is one of the leading causes of death for COAD patients, their 5-year survival rate remains unsatisfactory (3). Thus, identifying potential therapeutic targets and reliable prognostic biomarkers associated with tumor metastasis is crucial, as they may contribute to the timely diagnosis and precise treatment of COAD patients.

Most cancer cells can escape apoptosis, resulting in uncontrolled proliferation (4). Furthermore, they can become resistant to chemotherapeutic agents through mutated apoptotic programs (5, 6). As a result, understanding novel programmed cell death pathways is particularly essential for combating drug resistance. PANoptosis is an inflammatory programmed cell death dependent on the PANoptosome complex (7). It contains molecules essential for pyroptosis, apoptosis, and necroptosis that activate different cell death pathways (8). Therefore, even when other programmed cell death pathways are inhibited or mutated, PANoptosis can provide an alternative pathway to kill cancer cells and reduce the risk of acquiring drug resistance. Interferon regulatory factor 1 prevents CRC development by regulating PANoptosis (9). In addition, adenosine deaminase acting on RNA 1 inhibits ZBP1-mediated PANoptosis to promote CRC development (10). These findings suggest that PANoptosis is especially important for the progression of COAD, and identifying effective PANoptosis-associated biomarkers in COAD is paramount.

Long non-coding RNAs (lncRNAs) are generally defined as transcripts longer than 200 nucleotides that regulate cancer development and progression (11). They usually act as a competitive endogenous RNA (ceRNA) that regulates the ability of microRNAs (miRNAs) to inhibit target mRNA from translating into proteins (12, 13). Increasing evidence supports that lncRNAs participate in the progression of COAD, including cell proliferation, apoptosis, metastasis, and invasion (14). Few studies, however, have addressed the role of PANoptosis-associated lncRNAs in COAD. Thus, elucidating their role in COAD may improve our understanding of how it develops and help us find new treatment strategies against it.

In this study, The Cancer Genome Atlas (TCGA) and GEO databases were used to identify differential lncRNAs associated with metastasis and PANoptosis in COAD. Differentially expressed lncRNAs were used to a construct lncRNA-miRNA-mRNA network, and the functional and prognostic significance of the differentially expressed lncRNAs was further investigated by multiple bioinformatics analyses. In addition, we also analyzed the correlation between the expression of lncRNA SNHG7 in COAD and drug sensitivity. Finally, *in vitro* experiments demonstrated that lncRNA SNHG7 is involved in the progression of COAD. Our study provides a theoretical framework for identifying new therapeutic targets and prognostic biomarkers for COAD.

## Materials and Methods

### Data Collection

**Figure 1:**
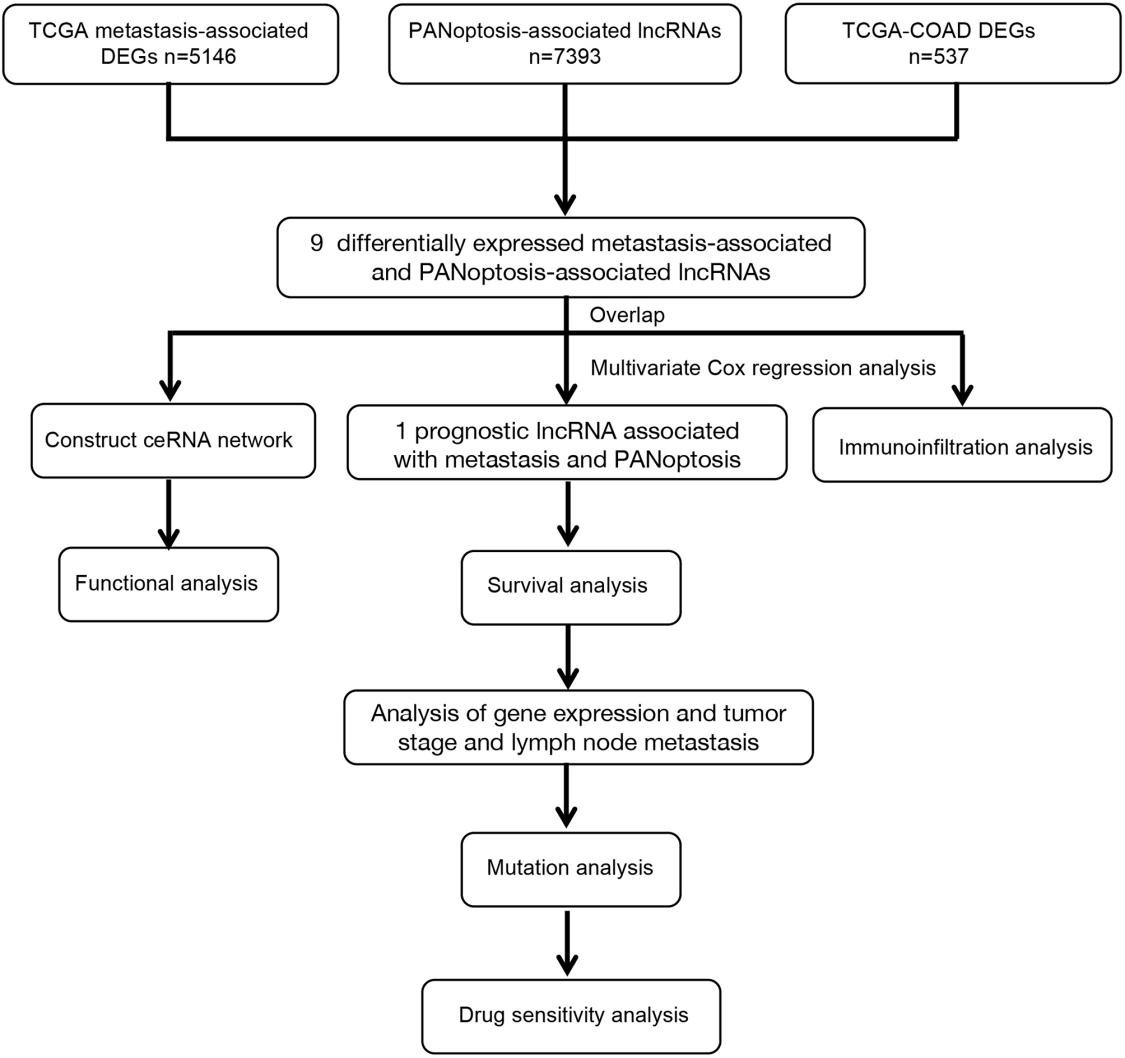
Workflow Diagram.

Transcriptomic and clinical data for COAD were retrieved from the TCGA database (https://portal.gdc.cancer.gov). Only samples of patients with complete clinical information were included in the study. Subsequent analyses were performed on 439 COAD and 39 adjacent normal samples. **Table 1** shows COAD patients’ characteristics and clinical data from TCGA. Metastasis-associated colon cancer genes were obtained from the GSE89393 transcriptome dataset from the GEO database (https://www.ncbi.nlm.nih.gov/geo/). Screening the TCGA-COAD and GSE89393 datasets for differentially expressed genes was done using the limma package in R with the screening criteria |Log_2_ FC| ≥ 0.5 and *P* < 0.05 (**Supplementary Tables 1** and **2**). Previous studies were used to narrow the gene list and extract 14 PANoptosis-associated genes, which are mainly PANoptosome components (15). PANoptosis-associated lncRNAs were identified according to the criterium where the absolute value of Pearson’s correlation coefficient is |R| > 0.3 and significance is *P* < 0.05 (**Supplementary Table 3** and **Figure 1**).

**Table 1:** Characteristics of TCGA-COAD database.

Characteristics	Tumor(N=439)	Adjacent normal(N=39)	Total(N=478)	pvalue	FDR
**Age**				0.3	1
<65	167(34.94%)	11(2.30%)	178(37.24%)		
≥65	272(56.90%)	28(5.86%)	300(62.76%)		
**Gender**				1	1
FEMALE	210(43.93%)	19(3.97%)	229(47.91%)		
MALE	229(47.91%)	20(4.18%)	249(52.09%)		
**T stage**				0.44	1
T1	9(1.88%)	0(0.0e+0%)	9(1.88%)		
T2	74(15.48%)	4(0.84%)	78(16.32%)		
T3	301(62.97%)	29(6.07%)	330(69.04%)		
T4	28(5.86%)	5(1.05%)	33(6.90%)		
T4a	18(3.77%)	0(0.0e+0%)	18(3.77%)		
T4b	8(1.67%)	1(0.21%)	9(1.88%)		
Tis	1(0.21%)	0(0.0e+0%)	1(0.21%)		
**N stage**				0.86	1
N0	256(53.56%)	27(5.65%)	283(59.21%)		
N1	71(14.85%)	5(1.05%)	76(15.90%)		
N1a	15(3.14%)	1(0.21%)	16(3.35%)		
N1b	15(3.14%)	0(0.0e+0%)	15(3.14%)		
N1c	2(0.42%)	0(0.0e+0%)	2(0.42%)		
N2	61(12.76%)	5(1.05%)	66(13.81%)		
N2a	8(1.67%)	0(0.0e+0%)	8(1.67%)		
N2b	11(2.30%)	1(0.21%)	12(2.51%)		
**M stage**				0.66	1
M0	328(68.62%)	27(5.65%)	355(74.27%)		
M1	52(10.88%)	7(1.46%)	59(12.34%)		
M1a	9(1.88%)	0(0.0e+0%)	9(1.88%)		
M1b	3(0.63%)	0(0.0e+0%)	3(0.63%)		
MX	47(9.83%)	5(1.05%)	52(10.88%)		
**Pathologic stage**				0.0099	0.07
Stage I	73(15.27%)	4(0.84%)	77(16.11%)		
Stage IA	1(0.21%)	0(0.0e+0%)	1(0.21%)		
Stage II	29(6.07%)	11(2.30%)	40(8.37%)		
Stage IIA	134(28.03%)	10(2.09%)	144(30.13%)		
Stage IIB	9(1.88%)	0(0.0e+0%)	9(1.88%)		
Stage IIC	1(0.21%)	0(0.0e+0%)	1(0.21%)		
Stage III	20(4.18%)	2(0.42%)	22(4.60%)		
Stage IIIA	8(1.67%)	0(0.0e+0%)	8(1.67%)		
Stage IIIB	59(12.34%)	4(0.84%)	63(13.18%)		
Stage IIIC	41(8.58%)	1(0.21%)	42(8.79%)		
Stage IV	45(9.41%)	6(1.26%)	51(10.67%)		
Stage IVA	17(3.56%)	1(0.21%)	18(3.77%)		
Stage IVB	2(0.42%)	0(0.0e+0%)	2(0.42%)		
**Survival status**				0.39	1
Alive	347(72.59%)	28(5.86%)	375(78.45%)		
Dead	92(19.25%)	11(2.30%)	103(21.55%)		

### Screening PANoptosis- and Metastasis-Associated lncRNAs in Colon Adenocarcinoma

The above datasets were analyzed sing the VennDiagram R package to identify lncRNA candidates. Heatmap showing the expression of 9 candidate lncRNAs in COAD and adjacent normal samples was generated with the Pheatmap R package. The R package ggplot2 was used to describe the correlation between the candidate lncRNAs and PANoptosis-associated genes. Sankey diagrams drawn by the “ggalluvial” R package were used to visualize the link between the lncRNA, PANoptosis-associated genes, and COAD prognosis.

### Constructing the lncRNA-miRNA-mRNA Network

MicroRNA targets bound to PANoptosis- and metastasis-associated lncRNAs were predicted with the lncBase Predicted v.2 database (https://dianalab.e-ce.uth.gr/tools). Subsequently, the miRDB database (http://mirdb.org/index.html) was used to predict mRNA targets of the identified miRNAs. Pearson correlation analysis where *P* < 0.05 was considered statistically significant was applied to predict miRNA and mRNA targets that co-express with lncRNAs. The lncRNA-miRNA-mRNA network was constructed and visualized using Cytoscape 3.9.0 software.

### Functional Enrichment Analysis of mRNAs in the ceRNA Network

Functional enrichment analysis was performed on 356 mRNAs co-expressed with lncRNAs in the lncRNA-miRNA-mRNA network. Gene Ontology (GO) and Kyoto Encyclopedia of Genes and Genomes (KEGG) analyses were conducted with the R package clusterProfiler.

### Immune Analysis of Candidate PANoptosis- and Metastasis-Associated lncRNAs

Single-sample gene set enrichment analysis (ssGSEA) in the R package GSVA was done to assess the association of candidate lncRNAs with 9 immune cells and immune cell enrichment scores for high and low lncRNA expression groups. The R package ESTIMATE was used to calculate the patients’ ESTIMATE, immune, and stromal scores to assess cellular components.

### Prognostic and Expression Analysis of lncRNA SNHG7 High- and Low-Risk Groups

Multivariate Cox regression analysis was used to explore the relationship between the candidate lncRNAs and the prognosis in COAD patients. We discovered a prognosis-related lncRNA (SNHG7) with a *P* < 0.05 criterium. Subsequently, the Kaplan-Meier survival curve for lncRNA SNHG7 high- and low-risk groups was generated using the R package survival. The UALCAN database (http://ualcan.path.uab.edu/) was utilized to create lncRNA SNHG7 expression box plots based on the patients’ sample types (Normal vs. Primary tumor), cancer stages (Normal vs. Stages I, II, III, and IV), and metastatic status (Normal vs. N_0_, N_1_, and N_2_).

### Mutational Analysis of SNHG7 in Colon Adenocarcinoma

The copy number variation (CNV) distribution of lncRNA SNHG7 in COAD tumor samples was determined with the Gene Set Cancer Analysis platform (http://bioinfo.life.hust.edu.cn/GSCA/#/). This platform was also utilized to visualize the correlation between SNHG7 CNV and expression in COAD and conclude the overall survival probability of three CNV types (Amplification, Deletion, and Wild type) for SNHG7 in COAD.

### Drug Sensitivity Analysis of SNHG7 in Colon Adenocarcinoma

Drug datasets were obtained from the CellMiner database (https://discover.nci.nih.gov/cellminer/home.do). The association of lncRNA SNHG7 expression with the drugs was analyzed by creating a scatter plot with the R package gglot2. Significant associations were defined with *P* < 0.05.

### Cell Culture and Plasmids

Human colorectal carcinoma cell line HCT116 was derived from the American-Type Culture Collection (ATCC). The cells were cultured in Dulbecco’s modified Eagle’s medium (Gibco, China) supplemented with 10% fetal bovine serum (Biological Industries, Israel) and maintained at 37°C with 5% CO_2_.

Lentiviral vector overexpressing lncRNA SNHG7 was generated by subcloning the SHNG7 cDNA sequence into the pLVX-EF1α-IRES-Puro plasmid (Clontech, Mountain View, CA, USA). Plasmid transfection was performed using ExFect Transfection Reagent (Vazyme, China) following the manufacturer’s instructions. The viral solution was collected to infect HCT116 cells, and colonies stably overexpressing SNHG7 were selected on puromycin.

### Reverse Transcription Quantitative Real-Time PCR

Total RNA was extracted from HCT116 cells using TRIzol reagent (Vazyme, China) with the manufacturer’s instructions. Total RNA was reverse transcribed into cDNA using a reverse transcription kit (Vazyme, China). Quantitative PCR assay was performed using MonAmp ChemoHS qPCR Mix (Monad, China). The primer sequences (F 5′-TTGCTGGCGTCTCGGTTAAT-3′, R 5′-GGAAGTC CATCACAGGCGAA-3′) for SNHG7 quantitation were taken from the literature (16).

### CCK-8 Assay

HCT116 cells stably overexpressing SNHG7 and controls were inoculated at a density of 1×10^4^ cells/ml into 96-well plates at 100 μl per well. After the cells fully adhered to the wall, their viability was measured at 0, 24, 48, and 72 h. Ten microliters of CCK-8 reagent (Vazyme, China) was added per well, and cells were incubated for 2 h at 37°C. The absorbance at 450 nm was measured using a Synergy 2 enzyme-labeling instrument (BioTek, USA). Finally, cell proliferation curves were plotted using GraphPad Prism (v 9.1.0).

### Colony Formation Assay

Approximately 100 control cells or HCT116 cells stably overexpressing SNHG7 were inoculated into 6-well plates and cultured for 2 weeks. The cells were fixed in 10% methanol for 15 min and stained with 0.1% crystal violet for 20 min. The number of visibly stained colonies was determined using ImageJ software (v 1.51, National Institutes of Health, USA).

### Cell Viability Assay

HCT116 cells were seeded into 96-well plates at a density of 2,000 cells/well. After 24 h incubation, the cells were treated with irinotecan at increasing concentrations for 72 h: 1 nM, 10 nM, 100 nM, 1 μM, 10 μM, and 100 μM. Ten microliters per well of CCK-8 reagent (Vazyme, China) was added to the cells, and they were incubated for 2 h at 37°C. The absorbance at 450 nm was measured using a Synergy 2 enzyme-labeling instrument (BioTek, USA). Finally, graphing was performed using GraphPad Prism (v 9.1.0) to obtain IC50 values of irinotecan in HCT116 cells.

### Statistical Analysis

Data were given as mean ± standard deviation. The Student’s *t*-test analyzed differences between groups. The correlation between the two variables was evaluated with Pearson’s correlation coefficient. Multivariate Cox regression analysis was performed to determine the prognostic indicators of overall survival. Data analysis was done in R (v 4.1.2) with statistical significance considered at *P* < 0.05.

## Results

### Identifying Metastasis-Associated and PANoptosis-Associated lncRNAs in Colon Adenocarcinoma

**Figure 2:**
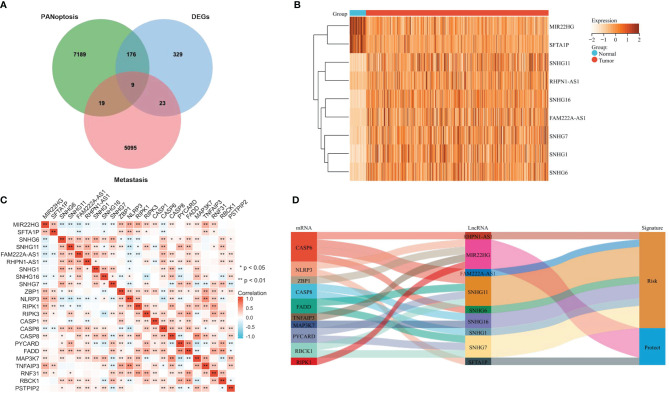
Identifying Metastasis- and PANoptosis-Related lncRNAs in COAD. **(A)** Venn diagram identifying metastasis-related genes, PANoptosis-related lncRNAs, and differentially expressed genes in TCGA-COAD. **(B)** Heatmap showing the differential expression of 9 candidate lncRNAs in COAD and adjacent normal tissues. **(C)** The correlation heatmap showing the correlation between 9 candidate lncRNAs and PANoptosis-related mRNAs. **(D)** Sankey diagram visualizes the degree of relationship between PANoptosis-related mRNAs, 9 candidate lncRNAs, and COAD prognosis.

The workflow diagram of the study is shown in **Figure 1**. We retrieved transcriptome profiles of 439 COAD and 39 adjacent normal samples from the TCGA database and metastatic colon cancer from the GEO database (dataset GSE89393). The metastasis-associated lncRNAs were obtained from the GSE89393 dataset. We screened the TCGA-COAD and GSE89393 datasets for differentially expressed genes with the R package limma and the screening criteria |Log_2_ FC| ≥ 0.5, *P* < 0.05. Reviewing the literature, we narrowed the list and identified 14 PANoptosis-related genes: ZBP1, NLRP3, RIPK1, RIPK3, CASP1, CASP6, CASP8, PYCARD, FADD, MAP3K7, TNFAIP3, RNF31, RBCK1, and PSTPIP2 (15). Next, using the criteria where |R| > 0.3 and *P* < 0.05, we performed a single-gene correlation analysis of the 14 genes to determine PANoptosis-associated lncRNAs. We found 9 lncRNAs (MIR22HG, SFTA1P, SNHG6, SNHG11, FAM222A-AS1, RHPN1-AS1, SNHG1, SNHG16, and SNHG7) present in all 3 groups (**Figure 2A**). The expression levels of these overlapping lncRNAs in COAD and adjacent normal samples were presented as a heatmap (**Figure 2B**). Among the overlapping lncRNAs, MIR22HG and SFTA1P had lower expression in tumors than in normal tissue. By contrast, SNHG6, SNHG11, FAM222A-AS1, RHPN1-AS1, SNHG1, SNHG16, and SNHG7 were highly expressed in tumors versus normal tissue. The correlation heatmap showed that the 9 candidates were closely related to PANoptosis-related mRNAs (**Figure 2C**). Furthermore, a Sankey diagram visualized the degree of correlation between PANoptosis-related mRNAs and the 9 candidate lncRNAs and demonstrated the relationship between them and the prognosis of COAD patients (**Figure 2D**).

### Constructing the lncRNA-miRNA-mRNA Network

**Figure 3:**
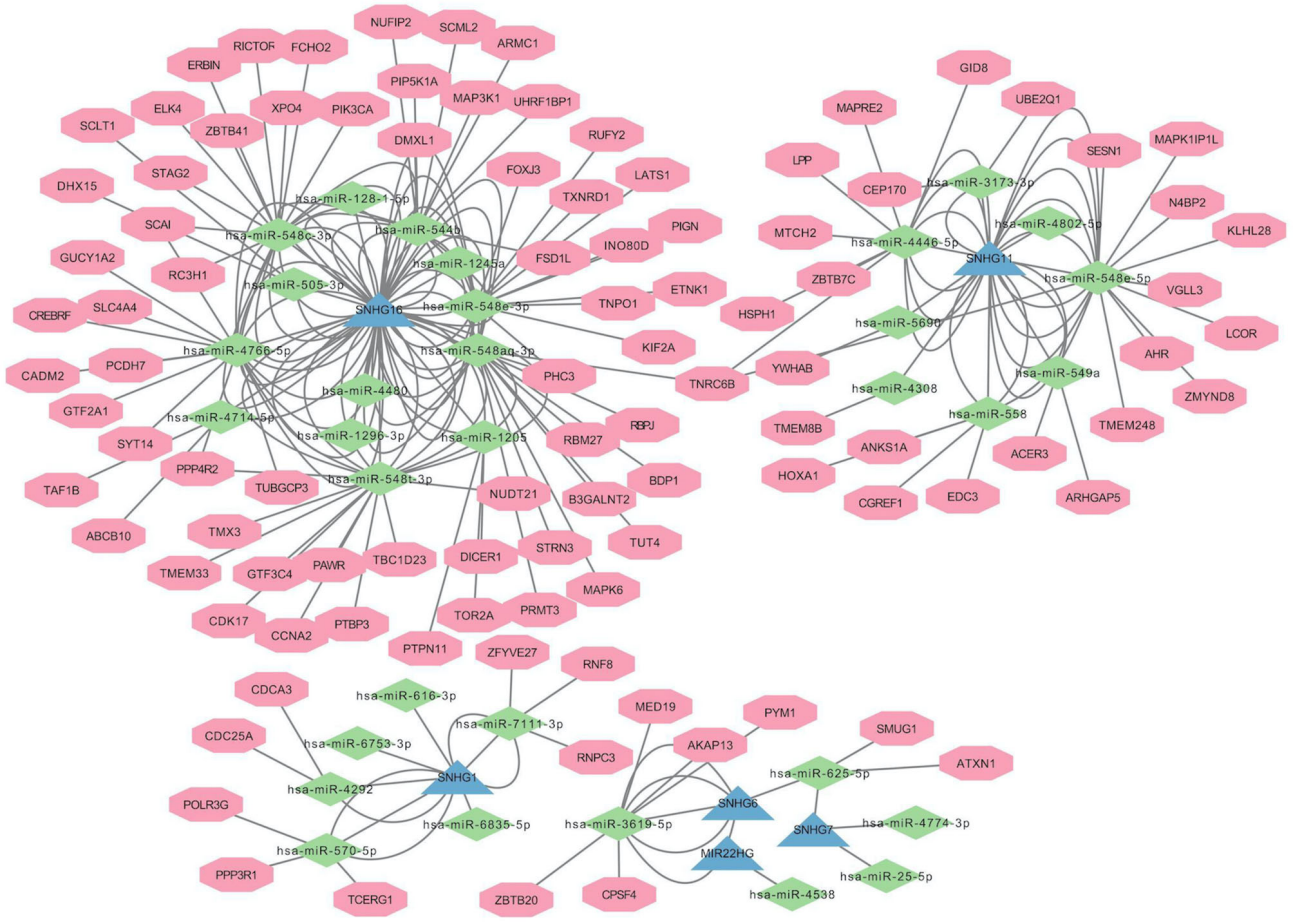
Construction of ceRNA Network of Candidate lncRNAs.

We constructed a lncRNA-miRNA-mRNA network (**Figure 3**) to explore the potential roles of the candidate lncRNAs in PANoptosis and COAD metastasis. Using the lncBase Predicted v.2 database, we predicted miRNA targets that interact with the candidate lncRNAs and screened out miRNAs co-expressed with lncRNAs in COAD (|R| > 0.3, *P* < 0.05). Subsequently, we identified the target miRNAs by taking the intersection between the two miRNA groups. Similarly, we used the miRDB database to assess mRNA targets of the miRNAs and identify the intersection with the mRNAs co-expressed with lncRNAs in COAD. Finally, we detected 6 lncRNAs, 32 miRNAs, and 356 mRNAs (**Table 2**) and visualized this lncRNA-miRNA-mRNA network with Cytoscape software (only the top ten mRNAs correlated with lncRNAs) (**Figure 3**).

**Table 2:** lncRNAs, miRNAs and mRNAs in the ceRNA Network.

lncRNAs	Binding miRNAs	Associated mRNAs
MIR22HG	hsa-miR-4538	
SNHG1	hsa-miR-616-3p, hsa-miR-570-5p, hsa-miR-6753-3p, hsa-miR-7111-3p, hsa-miR-4292, hsa-miR-6835-5p	TCERG1, POLR3G, PPP3R1, RNF8, RNPC3, ZFYVE27, CDC25A, CDCA3
SNHG6	hsa-miR-625-5p, hsa-miR-3619-5p	SMUG1, PYM1, ZBTB20, CPSF4, MED19, AKAP13
SNHG7	hsa-miR-625-5p, hsa-miR-25-5p, hsa-miR-4774-3p	ATXN1
SNHG11	hsa-miR-4802-5p, hsa-miR-5690, hsa-miR-549a, hsa-miR-3173-3p, hsa-miR-4308, hsa-miR-548e-5p, hsa-miR-558, hsa-miR-4446-5p	YWHAB, ARHGAP5, ACER3, UBE2Q1, CEP170, TMEM8B, N4BP2, AHR, SESN1, LCOR, KLHL28, VGLL3, ZMYND8, MAPK1IP1L, TNRC6B, TMEM248, HIPK1, SLC6A6, BLCAP, ESR1, CEP97, DMXL1, SPRR3, CLOCK, DOCK4, SETD7, ZBTB20, ELAVL1, SLC2A13, ARHGAP29, ZNF407, TOR1AIP2, KCTD9, HOXA1, CGREF1, ANKS1A, EDC3, GID8, LPP, MAPRE2, HSPH1, ZBTB7C, MTCH2
SNHG16	hsa-miR-548c-3p, hsa-miR-1245a, hsa-miR-548t-3p, hsa-miR-128-1-5p, hsa-miR-4480, hsa-miR-548aq-3p, hsa-miR-505-3p, hsa-miR-4714-5p, hsa-miR-1296-3p, hsa-miR-548e-3p, hsa-miR-544b, hsa-miR-1205, hsa-miR-4766-5p	ZBTB41, STAG2, XPO4, ERBIN, RICTOR, PIK3CA, SCAI, FCHO2, ELK4, RC3H1, SEC23IP, SPTY2D1, UBN2, KDM7A, NAPEPLD, ANGEL2, ZDHHC21, ARHGEF12, WDR43, NAMPT, GPAM, AFF4, DICER1, PHIP, RFX3, STRN, PDS5A, VCPIP1, CREB1, BRWD3, IKZF5, G3BP2, TMTC3, FOXN2, PTAR1,ZBTB21, TMEM41B, MMD, TNPO1, LIN7C, RLIM, TM9SF3, DDX46, ZNF148, GAB1, KLHL28, SSX2IP, FZD3, FBXO45, CRLF3, PIK3C2A, PRKAA1, SPRED1, ERCC6, REV1, KPNA4, PPP1CB, ARPP19, CLDND1, BTBD7, ZNF652, BBX, PURA, ATAD2B, MAPK8, INO80D, SPIN4, GNPNAT1, UBE2W,PARD6B, LRP6, SETD7, TMEM106B, SYDE2, REST, PXYLP1, TMED5, PTPN4, TRPM7, WDR36, PDK1, KIF21A, TMEM168, LRRTM2, SLC4A7, HNRNPU, DTWD2, SP3, SLC39A9, JMY, SF3B1, SMC5, PAK2, ATF1, CPNE3, NEDD1, NEK1, CARF, ITCH, CHRNA5, CCDC6, NUS1, PURB, SUZ12, TRIP11,RPGRIP1L, TTC39C, HS2ST1, ACER3, MTX3, RFX7, AP3M1, MYBL1, MAP3K2, ATXN3, GPR63, STXBP5, NEMP1, MTMR12, SIRT1, TMEM64, ERI2, TBL1XR1, SGPP1, MPP6, ZNF281, RAB11FIP2, USP45, CCDC62, GTF2E1, PPAT, CNOT6, DBR1, IRAK1BP1, KIAA0825, TAOK1, FAM210A, GABPA,USP13, MAPK1IP1L, ZBTB20, TNRC6B, FSD1L, MKLN1, ATP13A3, TMX3, MBTD1, ATF6, LRRC58, CNOT6L, NEMP2, FAM122A, PTBP3, CDK6, ARHGAP11A, EEA1, ELMOD2, PHC3, ARID2, CCDC43, C5orf51, SERBP1, GLCE, ABHD17B, CCNA2, TBC1D23, TMEM33, PAWR, CDK17, NUDT21, GTF3C4,PPP4R2, GRPEL2, RNF169, SGO2, NRAS, SLC25A36, RAB33B, RNFT1, PPP2R1B, MOB4, NUFIP2, LARP4, USP46, MIB1, STK38L, ZBTB39, PAPOLA, UBR2, SMAD5, RBPJ, PRMT3, BDP1, TUT4, STRN3, B3GALNT2, RBM27, MAPK6, TET2, HNRNPR, GTF2A1, RPS6KA3, MED13, SYNCRIP, NAB1,PDS5B, AZIN1, ETNK1, APPBP2, RSBN1, PTPDC1, ZNF326, KANSL1L, ACVR2B, NAA50, CD47, RECQL, YIPF4, FMNL2, MOB1B, C18orf25, CDYL, CCNT1, TGFBR1, PIGN, SPICE1, SLC18B1, PPP2R5E, USP53, MMGT1, ZBTB44, ARAP2, ADAM17, PHACTR2, ZNF24, DENND1B, SCYL2, PUM2,PRKAR1A, GNAI3, OSBPL8, DHX15, SCLT1, ABCB10, TAF1B, KIF2A, RUFY2, TXNRD1, LATS1, FOXJ3, SMAD4, FAM149B1, ZNF91, DR1, RAD51AP1, KLHL15, USP38, DSG2, ZNF280C, IPMK, TRAPPC8, EIF4E, HERC4, PAIP1, SCML2, PIP5K1A, MAP3K1, DMXL1, UHRF1BP1, ARMC1, TOR2A,PTPN11, SYT14, SLC4A4, CREBRF, CADM2, TUBGCP3, GUCY1A2, PCDH7, STAM2, CLVS2, GPBP1, GNB4, BOD1L1, MEIOC, C14orf28, ZNF236, VEPH1, VGLL3, HCN1, MCTP2, FAM126B, POLR2M, KALRN, MECP2, CLGN, C6orf62, ZBTB11, ELF1, AMMECR1, SEMA3A, SLC30A4, SLC18A1, CBX5,RFXAP, DPYS, TRIM13	

### Functional Enrichment Analysis of lncRNA-related mRNAs

**Figure 4:**
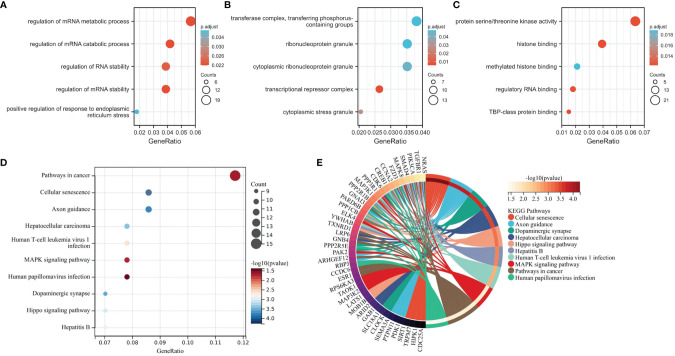
Functional and Pathway Enrichment Analysis of mRNAs in the lncRNA-miRNA-mRNA Network. **(A–C)** The mRNAs associate with three GO categories: biological process, cellular component, and molecular function. The most significant GO terms for lncRNA-related mRNAs are regulation of mRNA metabolic process, transferase complex, transferring phosphorus-containing groups, and protein serine/threonine kinase activity. **(D, E)** KEGG pathway analysis reveals mRNA-enriched associated signaling pathways. The most enriched KEGG pathway for lncRNA-associated mRNAs is the cancer.

To explore the biological functions and signal transduction pathways of the candidate lncRNAs, we performed GO and KEGG analysis on 356 lncRNA-related mRNAs in the lncRNA-miRNA-mRNA network. GO analysis showed these lncRNA-related mRNAs were primarily associated with different significant GO terms: regulation of mRNA metabolic process, transferase complex, transferring phosphorus-containing groups, and protein serine/threonine kinase activity (**Figures 4A–C**). The pathway enrichment analysis revealed that mRNAs were mainly enriched in tumors and associated with cellular senescence (**Figures 4D, E**).

### Immune Analysis of Candidate lncRNA

**Figure 5:**
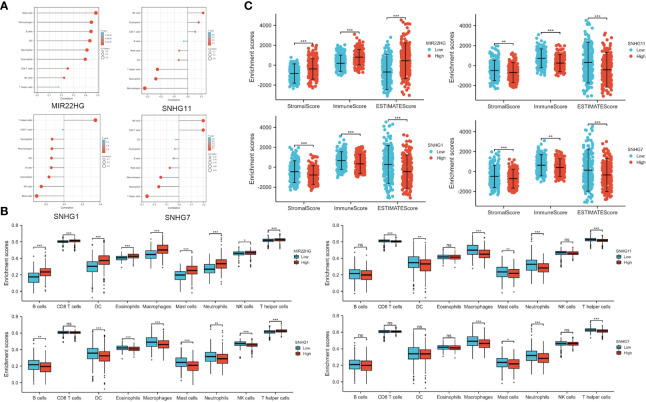
Immune Infiltration Analysis of Candidate PANoptosis- and Metastasis-Related lncRNAs. **(A)** Correlation of candidate lncRNAs with different tumor-infiltrating immune cells. **(B)** Immune cell infiltration in high and low lncRNA expression groups. **(C)** Comparison of ESTIMATE, immune, and stromal scores in candidate lncRNA high and low expression groups. *P < 0.05; **P < 0.01; ***P < 0.001; ns *P* > 0.05 (ns, not significant).

PANoptosis plays a crucial role in developing the tumor immune microenvironment (15). We used ssGSEA method to elucidate the correlation of candidate lncRNA expression with immune cell infiltration in COAD and define immune cell enrichment scores in high and low lncRNA expression groups. The data showed a correlation between candidate lncRNA expression and various tumor-infiltrating cells. For instance, lncRNAs MIR22HG and SFTA1P significantly positively correlated with macrophages, neutrophils, dendritic cells, and mast cells, whereas the remaining seven lncRNAs negatively correlated with most tumor-infiltrating cells, including B lymphocyte (**Figure 5A** and **Supplementary Figure 1A**). Moreover, there were multiple immune cell enrichment differences between the high and low lncRNA expression groups. For example, tumor-killing immune cells in the high expression group of SNHG16, SNHG6, SNHG11, FAM222A-AS1, RHPN1-AS1, SNHG1, and SNHG7 were significantly lower than those in the low (**Figure 5B** and **Supplementary Figure 1B**). We also used ESTIMATE, immune, and stromal scores to evaluate the tumor immune microenvironment in COAD. All 3 scores of the high expression group of SNHG6, SNHG11, FAM222A-AS1, RHPN1-AS1, SNHG1, and SNHG7 were lower than those of the low. By contrast, the scores of the low expression group of MIR22HG and SFTA1P were lower than those of the high (**Figure 5C** and **Supplementary Figure 1C**).

### Survival, Expression, and Mutational Analysis of SNHG7 in COAD

**Figure 6:**
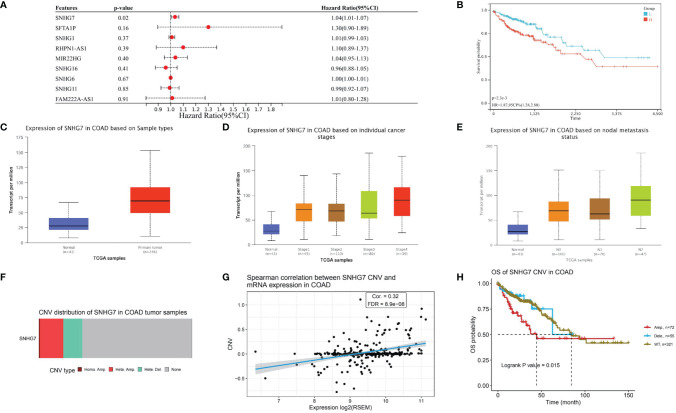
Survival, Expression, and Mutational Analysis of lncRNA SNHG7 in COAD. **(A)** Forest plot showing the results of multivariate Cox regression analysis of 9 lncRNAs and overall survival in COAD patients. **(B)** Kaplan-Meier survival analysis of SNHG7. **(C)** SNHG7 expression in COAD. **(D, E)** The relationship between SNHG7 expression in COAD with tumor stage and lymph node metastasis. **(F)** Copy number variation (CNV) type distribution of SNHG7 in COAD. **(G)** Correlation between SNHG7 CNV and expression in COAD **(H)** Overall survival of SNHG7 CNV in COAD.

To explore the relationship of the candidate lncRNAs with patient prognosis, we used multivariate Cox regression analysis to identified one prognostic lncRNA (SNHG7) associated with PANoptosis and COAD metastasis (**Figure 6A**). It also had a significantly poor prognosis in the high-risk group compared with the low (**Figure 6B**). To further confirm the relationship of lncRNA SNHG7 with metastasis and prognosis, we analyzed its expression in different groups. We found it gradually increased with higher tumor stage and increased lymph node metastasis (**Figures 6C–E**). Mutational analysis revealed that amplification is the leading mutation in the SNHG7 gene (**Figure 6F**). Moreover, expression levels of lncRNA SNHG7 positively correlated with the degree of CNV in COAD (**Figure 6G**). Finally, overall survival was significantly lower in patients with predominantly SNHG7 amplification mutations (**Figure 6H**). These results indicate high expression of PANoptosis-associated lncRNA SNHG7 predicts poor prognosis in COAD.

### SNHG7 Contributes to COAD Cell Proliferation and Irinotecan Resistance

**Figure 7:**
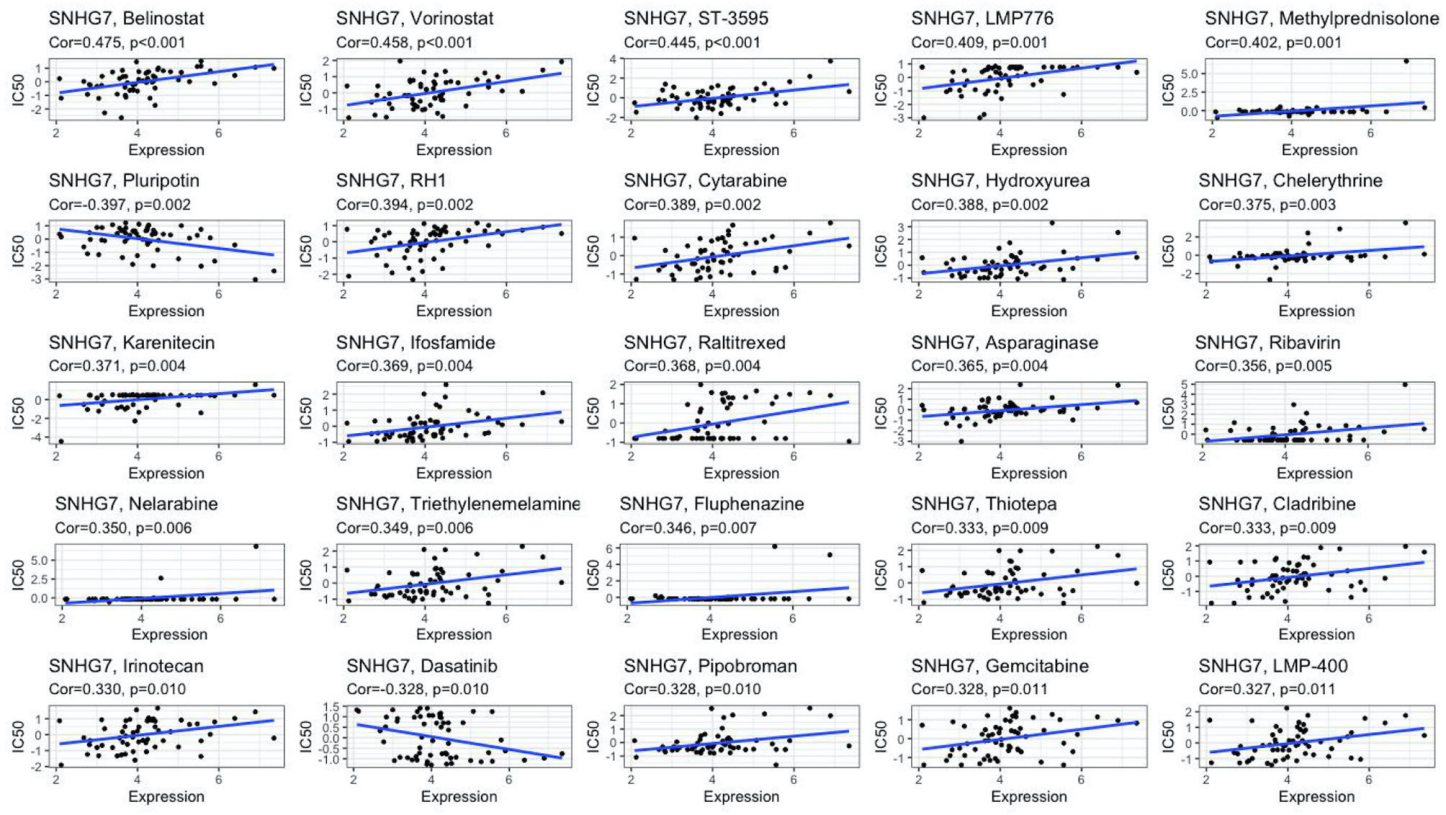
Drug sensitivity analysis of SNHG7.

We performed *in vitro* experiments to investigate whether lncRNA SNHG7 plays a role in the progression of COAD. SNHG7 expression is relatively low in the HCT116 cell line (16, 17). Thus, we overexpressed SNHG7 in HCT116 cells and verified its overexpression with RT-qPCR (**Supplementary Figure 2A**). CCK-8 and clone formation assays showed that SNHG7 overexpression promotes proliferation of HCT116 cells (**Supplementary Figures 2B** and **C**), confirming lncRNA SNHG7 stimulates COAD progression. Since tumor metastasis and anti-apoptosis often accompany the development of drug resistance (18, 19), we explored whether SNHG7 is associated with drug resistance. Drug datasets were obtained from the CellMiner database, and the association of SNHG7 expression with more than 20,000 compounds was analyzed by creating a scatter plot with the R package gglot2. **Figure 7** and **Table 3** show the relationship between drug sensitivity and SNHG7 expression for 25 compounds correlating most closely with SNHG7 expression. Overall, drug sensitivity analysis showed that the expression of SNHG7 positively correlated with 50% inhibition concentration (IC50) of most drugs. Irinotecan is a first-line chemotherapeutic drug for treating patients with metastatic COAD (3, 20, 21), and its IC50 values positively correlate with SNHG7 expression. These results suggest that lncRNA SNHG7 may be involved in chemo-resistance in COAD. Hence, we subsequently treated SNHG7-overexpressing HCT116 cells with irinotecan for 72 h and subjected the treated cells to a cell viability assay. The IC50 value of the overexpression group (3,897 nM) was 2,639 nM higher than that of the empty vector group (1,258 nM) (**Supplementary Figure 2D**). This finding indicates that overexpressing SNHG7 in HCT116 cells enhances their resistance to irinotecan and supports the results of the bioinformatics analysis. In summary, the expression of lncRNA SNHG7 in COAD affects the proliferation of COAD cells and the therapeutic effect of chemotherapeutic drugs. Therefore, SNHG7 may be key to solving problems related to poor therapeutic effects in COAD patients.

**Table 3:** Drug Sensitivity Analysis of SNHG7.

Gene	Drug	cor	pvalue
SNHG7	Belinostat	0.475150756	0.000124941
SNHG7	Vorinostat	0.458034671	0.000233285
SNHG7	ST-3595	0.44524744	0.000364234
SNHG7	LMP776	0.409005076	0.00117581
SNHG7	Methylprednisolone	0.401629543	0.001469495
SNHG7	Pluripotin	-0.396574183	0.001707192
SNHG7	RH1	0.394164857	0.001832149
SNHG7	Cytarabine	0.389406688	0.002103212
SNHG7	Hydroxyurea	0.388341558	0.002168588
SNHG7	Chelerythrine	0.374746552	0.00317741
SNHG7	Karenitecin	0.370615145	0.003557304
SNHG7	Ifosfamide	0.368587346	0.003758079
SNHG7	Raltitrexed	0.367598446	0.00385958
SNHG7	Asparaginase	0.364779387	0.004162362
SNHG7	Ribavirin	0.35637975	0.00519255
SNHG7	Nelarabine	0.350020389	0.006115555
SNHG7	Triethylenemelamine	0.349079912	0.006263608
SNHG7	Fluphenazine	0.346037147	0.006764353
SNHG7	Thiotepa	0.333471406	0.009222025
SNHG7	Cladribine	0.332826742	0.009366744
SNHG7	Irinotecan	0.330403599	0.009928501
SNHG7	Dasatinib	-0.328392077	0.010416805
SNHG7	Pipobroman	0.328083936	0.010493415
SNHG7	Gemcitabine	0.327504385	0.010638828
SNHG7	LMP-400	0.327348563	0.010678221

## Discussion

PANoptosis is a recently discovered type of programmed cell death that promotes tumor cell death and perhaps reduces the effect of aberrant apoptotic pathways on chemo-resistance in tumors (5, 6). However, few studies on the relationship between PANoptosis and COAD exist. Metastases are one of the leading causes of COAD-related deaths. Abundant evidence suggests that the abnormal expression of lncRNAs affects the proliferation, apoptosis, drug resistance, migration, and invasion of COAD cells (22–27). How lncRNAs influence PANoptosis is yet unknown. At present, we only know that lncRNA TUG1 likely acts on FADD, an PANoptosis initiator, but the exact mechanism remains unclear (28). Therefore, we aimed to link PANoptosis, COAD metastasis, and lncRNAs to uncover the specific regulatory roles of PANoptosis- and COAD metastasis-related lncRNAs in COAD.

Our study identified 9 lncRNAs related to PANoptosis and COAD metastasis through a comprehensive analysis of publicly available transcriptome datasets. Subsequently, we constructed a ceRNA network of candidate lncRNAs in COAD. To better grasp the molecular functions of aberrantly expressed lncRNAs, we analyzed 356 mRNAs associated with lncRNAs in the network. Pathway enrichment analyses showed that most network pathways were enriched in cancer and associated with tumor proliferation, apoptosis, drug resistance, invasion, and migration, including the Hippo and MAPK signaling (29–31). Immune cells in the tumor immune microenvironment play key roles in tumor control and immunity. PANoptosis is also associated with the tumor microenvironment (6). Immune infiltration analysis uncovered that 9 lncRNAs were significantly associated with immune infiltration. The low expression group of SNHG16, SNHG6, SNHG11, FAM222A-AS1, RHPN1-AS1, SNHG1, and SNHG7 was accompanied by more immune cell infiltration, further supporting that PANoptosis plays a crucial role in the tumor immune microenvironment. LncRNAs SNHG16 and SNHG1 have been proven to regulate the cancer immune microenvironment. Ni et al. found that SNHG16 activates the TGF-β1/SMAD5 pathway by targeting miR-16-5p, causing the transformation of γδ1 T cells into the CD73^+^ immunosuppressive subtype (32). Likewise, SNHG1 participates in immune escape by regulating T cells in renal cell carcinoma and breast cancer (33, 34).

Multivariate Cox regression analysis showed that LncRNA SNHG7 considerably correlated with the prognosis of COAD patients, and its high expression significantly reduced their survival rate. The expression of SNHG7 also significantly positively correlated with the tumor stage and lymph node metastasis in COAD. We demonstrated that SNHG7 promotes COAD progression by enhancing cell proliferation. And previous experiments have fully proved that SNHG7 can also affect tumor cell apoptosis, invasion and migration in COAD. For example, SNHG7 binds miR-216b and promotes CRC proliferation and liver metastasis by upregulating GALNT1 (17). Inhibiting K-ras/ERK/cyclin D1 pathway by silencing SNHG7 reduces CRC proliferation and promotes apoptosis (35). In addition, SNHG7 promotes tumor chemo-resistance in various tumors. For instance, exosome-mediated transfer of SNHG7 enhances docetaxel resistance in lung adenocarcinoma (36). Similarly, it increases trastuzumab resistance in breast cancer cells *via* regulating miR-186 (37). Our drug sensitivity analysis found a correlation between the expression of SNHG7 and the first-line chemotherapy drug irinotecan, typically given for treating metastatic COAD. High expression of SNHG7 enhances tumor resistance to irinotecan. To validate, we examined IC50 values of irinotecan in COAD cell lines overexpressing SNHG7 and control cells. Indeed, higher IC50 values in the overexpressing cells versus the control were consistent with our assumption that SNHG7 contributes to irinotecan resistance in COAD.

Although our study indicates that PANoptosis- and COAD metastasis-related lncRNA SNHG7 has decisive regulatory roles in COAD, it has limitations. The sample size is limited without any supporting clinical samples, which may cause deviations. Moreover, the underlying mechanism by which SNHG7 promotes COAD progression requires further investigation.

## Conclusion

In conclusion, we carried out a comprehensive and systematic bioinformatics analysis of COAD patient samples and demonstrated that the occurrence and progression of COAD are closely related to PANoptosis. We also identified PANoptosis-related lncRNA SNHG7 associated with COAD metastasis, chemo-resistance, and prognosis. Therefore, we suggest lncRNA SNHG7 as a potential prognostic biomarker and therapeutic target for COAD.

## Data Availability Statement

The original contributions presented in the study are included in the article/**Supplementary Material**. Further inquiries can be directed to the corresponding author.

## Author Contributions

YJ contributed conception, administration of this study and revised this manuscript. JH contributed equally in analysis and wrote the manuscript. SJ, LL, HH, YL, LC helped in data collection and organization. All authors have read and agreed to the final manuscript.

## Funding

This work was supported by the National Natural Science Foundation of China [81802785 (YJ), 82100490 (LC)], the Hunan Provincial Natural Science Foundation of China [2020JJ5382 (YJ), 2020JJ5381 (LC)], Scientific Research Program of Hunan Provincial Health Commission [202202065445 (YJ)].

## Conflict of Interest

The authors declare that the research was conducted in the absence of any commercial or financial relationships that could be construed as a potential conflict of interest.

## Publisher’s Note

All claims expressed in this article are solely those of the authors and do not necessarily represent those of their affiliated organizations, or those of the publisher, the editors and the reviewers. Any product that may be evaluated in this article, or claim that may be made by its manufacturer, is not guaranteed or endorsed by the publisher.

## Abbreviations

CRC, colorectal cancer; COAD, colon adenocarcinoma; lncRNA, long noncoding RNA; ceRNA, competitive endogenous RNA; miRNA, microRNA; TCGA, the Cancer Genome Atlas; GO, Gene Ontology; KEGG, Kyoto Encyclopedia of Genes and Genomes; ssGSEA, Single-sample gene set enrichment analysis; CNV, copy number variation; IC50, 50% inhibition concentration.

## References

[1] Sung H Ferlay J Siegel RL Laversanne M Soerjomataram I Jemal A. Global Cancer Statistics 2020: Globocan Estimates of Incidence and Mortality Worldwide for 36 Cancers in 185 Countries. CA Cancer J Clin (2021) 71(3):209–49. doi: 10.3322/caac.21660 33538338

[2] Li J. Digestive Cancer Incidence and Mortality Among Young Adults Worldwide in 2020: A Population-Based Study. World J Gastrointest Oncol (2022) 14(1):278–94. doi: 10.4251/wjgo.v14.i1.278 PMC8790416 35116117

[3] Kuipers EJ Grady WM Lieberman D Seufferlein T Sung JJ Boelens PG. Colorectal Cancer. Nat Rev Dis Primers (2015) 1:15065. doi: 10.1038/nrdp.2015.65 27189416 PMC4874655

[4] Mohammad RM Muqbil I Lowe L Yedjou C Hsu HY Lin LT. Broad Targeting of Resistance to Apoptosis in Cancer. Semin Cancer Biol (2015) 35 Suppl:S78–S103. doi: 10.1016/j.semcancer.2015.03.001 25936818 PMC4720504

[5] Malireddi RKS Tweedell RE Kanneganti TD. Panoptosis Components, Regulation, and Implications. Aging (Albany NY) (2020) 12(12):11163–4. doi: 10.18632/aging.103528 PMC7343493 32575071

[6] Jiang M Qi L Li L Wu Y Song D Li Y. Caspase-8: A Key Protein of Cross-Talk Signal Way in "Panoptosis" in Cancer. Int J Cancer (2021) 149(7):1408–20. doi: 10.1002/ijc.33698 34028029

[7] Wang Y Kanneganti TD. From Pyroptosis, Apoptosis and Necroptosis to Panoptosis: A Mechanistic Compendium of Programmed Cell Death Pathways. Comput Struct Biotechnol J (2021) 19:4641–57. doi: 10.1016/j.csbj.2021.07.038 PMC8405902 34504660

[8] Christgen S Zheng M Kesavardhana S Karki R Malireddi RKS Banoth B. Identification of the Panoptosome: A Molecular Platform Triggering Pyroptosis, Apoptosis, and Necroptosis (Panoptosis). Front Cell Infect Microbiol (2020) 10:237. doi: 10.3389/fcimb.2020.00237 32547960 PMC7274033

[9] Karki R Sharma BR Lee E Banoth B Malireddi RKS Samir P. Interferon Regulatory Factor 1 Regulates Panoptosis to Prevent Colorectal Cancer. JCI Insight (2020) 5(12):e136720. doi: 10.1172/jci.insight.136720 PMC7406299 32554929

[10] Karki R Sundaram B Sharma BR Lee S Malireddi RKS Nguyen LN. Adar1 Restricts Zbp1-Mediated Immune Response and Panoptosis to Promote Tumorigenesis. Cell Rep (2021) 37(3):109858. doi: 10.1016/j.celrep.2021.109858 34686350 PMC8853634

[11] Huarte M. The Emerging Role of Lncrnas in Cancer. Nat Med (2015) 21(11):1253–61. doi: 10.1038/nm.3981 26540387

[12] Salmena L Poliseno L Tay Y Kats L Pandolfi PP. A Cerna Hypothesis: The Rosetta Stone of a Hidden Rna Language? Cell (2011) 146(3):353–8. doi: 10.1016/j.cell.2011.07.014 PMC3235919 21802130

[13] Zhao Y Zhang H Ju Q Li X Zheng Y. Comprehensive Analysis of Survival-Related Lncrnas, Mirnas, and Mrnas Forming a Competing Endogenous Rna Network in Gastric Cancer. Front Genet (2021) 12:610501. doi: 10.3389/fgene.2021.610501 33737947 PMC7960915

[14] Chen S Shen X. Long Noncoding Rnas: Functions and Mechanisms in Colon Cancer. Mol Cancer (2020) 19(1):167. doi: 10.1186/s12943-020-01287-2 33246471 PMC7697375

[15] Samir P Malireddi RKS Kanneganti TD. The Panoptosome: A Deadly Protein Complex Driving Pyroptosis, Apoptosis, and Necroptosis (Panoptosis). Front Cell Infect Microbiol (2020) 10:238. doi: 10.3389/fcimb.2020.00238 32582562 PMC7283380

[16] Li Y Zeng C Hu J Pan Y Shan Y Liu B. Long Non-Coding Rna-Snhg7 Acts as a Target of Mir-34a to Increase Galnt7 Level and Regulate Pi3k/Akt/Mtor Pathway in Colorectal Cancer Progression. J Hematol Oncol (2018) 11(1):89. doi: 10.1186/s13045-018-0632-2 29970122 PMC6029165

[17] Shan Y Ma J Pan Y Hu J Liu B Jia L. Lncrna Snhg7 Sponges Mir-216b to Promote Proliferation and Liver Metastasis of Colorectal Cancer Through Upregulating Galnt1. Cell Death Dis (2018) 9(7):722. doi: 10.1038/s41419-018-0759-7 29915311 PMC6006356

[18] Norouzi S Gorgi Valokala M Mosaffa F Zirak MR Zamani P Behravan J. Crosstalk in Cancer Resistance and Metastasis. Crit Rev Oncol Hematol (2018) 132:145–53. doi: 10.1016/j.critrevonc.2018.09.017 30447920

[19] Kaufmann SH Vaux DL. Alterations in the Apoptotic Machinery and Their Potential Role in Anticancer Drug Resistance. Oncogene (2003) 22(47):7414–30. doi: 10.1038/sj.onc.1206945 14576849

[20] Koopman M Antonini NF Douma J Wals J Honkoop AH Erdkamp FL. Sequential Versus Combination Chemotherapy With Capecitabine, Irinotecan, and Oxaliplatin in Advanced Colorectal Cancer (Cairo): A Phase III Randomised Controlled Trial. Lancet (2007) 370(9582):135–42. doi: 10.1016/S0140-6736(07)61086-1 17630036

[21] Van Cutsem E Cervantes A Nordlinger B Arnold D Group EGW. Metastatic Colorectal Cancer: Esmo Clinical Practice Guidelines for Diagnosis, Treatment and Follow-Up. Ann Oncol (2014) 25(Suppl 3):iii1–9. doi: 10.1093/annonc/mdu260 25190710

[22] Yang H Wang S Kang YJ Wang C Xu Y Zhang Y. Long Non-Coding Rna Snhg1 Predicts a Poor Prognosis and Promotes Colon Cancer Tumorigenesis. Oncol Rep (2018) 40(1):261–71. doi: 10.3892/or.2018.6412 PMC6059747 29749530

[23] Yue B Cai D Liu C Fang C Yan D. Linc00152 Functions as a Competing Endogenous Rna to Confer Oxaliplatin Resistance and Holds Prognostic Values in Colon Cancer. Mol Ther (2016) 24(12):2064–77. doi: 10.1038/mt.2016.180 PMC5167786 27633443

[24] Chen S Bu D Ma Y Zhu J Chen G Sun L. H19 Overexpression Induces Resistance to 1,25(Oh)2d3 by Targeting Vdr Through Mir-675-5p in Colon Cancer Cells. Neoplasia (2017) 19(3):226–36. doi: 10.1016/j.neo.2016.10.007 PMC5300698 28189050

[25] Ni X Ding Y Yuan H Shao J Yan Y Guo R. Long Non-Coding Rna Zeb1-As1 Promotes Colon Adenocarcinoma Malignant Progression *Via* Mir-455-3p/Pak2 Axis. Cell Prolif (2020) 53(1):e12723. doi: 10.1111/cpr.12723 31828845 PMC6985675

[26] Dong Y Wei MH Lu JG Bi CY. Long Non-Coding Rna Hulc Interacts With Mir-613 to Regulate Colon Cancer Growth and Metastasis Through Targeting Rtkn. BioMed Pharmacother (2019) 109:2035–42. doi: 10.1016/j.biopha.2018.08.017 30551459

[27] Tian W Du Y Ma Y Gu L Zhou J Deng D. Malat1-Mir663a Negative Feedback Loop in Colon Cancer Cell Functions Through Direct Mirna-Lncrna Binding. Cell Death Dis (2018) 9(9):857. doi: 10.1038/s41419-018-0925-y 30154407 PMC6113222

[28] Wang S Hou Y Xing N Meng X Zhang Y Wang X. Identification of a Novel Prognostic Signature Related to Panoptosis and Its Regulatory Mechanism as Well as Targeted Treatment of Active Ingredients and Traditional Chinese Medicine in Lung Adenocarcinoma. Pharmacol Res - Modern Chin Med (2022) 2:100069. doi: 10.1016/j.prmcm.2022.100069

[29] Misra JR Irvine KD. The Hippo Signaling Network and Its Biological Functions. Annu Rev Genet (2018) 52:65–87. doi: 10.1146/annurev-genet-120417-031621 30183404 PMC6322405

[30] Zanconato F Cordenonsi M Piccolo S. Yap/Taz at the Roots of Cancer. Cancer Cell (2016) 29(6):783–803. doi: 10.1016/j.ccell.2016.05.005 27300434 PMC6186419

[31] McCubrey JA Steelman LS Chappell WH Abrams SL Wong EW Chang F. Roles of the Raf/Mek/Erk Pathway in Cell Growth, Malignant Transformation and Drug Resistance. Biochim Biophys Acta (2007) 1773(8):1263–84. doi: 10.1016/j.bbamcr.2006.10.001 PMC2696318 17126425

[32] Ni C Fang QQ Chen WZ Jiang JX Jiang Z Ye J. Breast Cancer-Derived Exosomes Transmit Lncrna Snhg16 to Induce Cd73+Gammadelta1 Treg Cells. Signal Transduct Target Ther (2020) 5(1):41. doi: 10.1038/s41392-020-0129-7 32345959 PMC7188864

[33] Tian P Wei JX Li J Ren JK Yang JJ. Lncrna Snhg1 Regulates Immune Escape of Renal Cell Carcinoma by Targeting Mir-129-3p to Activate Stat3 and Pd-L1. Cell Biol Int (2021) 45(7):1546–60. doi: 10.1002/cbin.11595 33739543

[34] Pei X Wang X Li H. Lncrna Snhg1 Regulates the Differentiation of Treg Cells and Affects the Immune Escape of Breast Cancer *Via* Regulating Mir-448/Ido. Int J Biol Macromol (2018) 118(Pt A):24–30. doi: 10.1016/j.ijbiomac.2018.06.033 29886172

[35] Liu KL Wu J Li WK Li NS Li Q Lao YQ. Lncrna Snhg7 Is an Oncogenic Biomarker Interacting With Microrna-193b in Colon Carcinogenesis. Clin Lab (2019) 65(11):2199–204. doi: 10.7754/Clin.Lab.2019.190501 31721543

[36] Zhang K Chen J Li C Yuan Y Fang S Liu W. Exosome-Mediated Transfer of Snhg7 Enhances Docetaxel Resistance in Lung Adenocarcinoma. Cancer Lett (2022) 526:142–54. doi: 10.1016/j.canlet.2021.10.029 34715254

[37] Zhang H Zhang XY Kang XN Jin LJ Wang ZY. Lncrna-Snhg7 Enhances Chemotherapy Resistance and Cell Viability of Breast Cancer Cells by Regulating Mir-186. Cancer Manag Res (2020) 12:10163–72. doi: 10.2147/CMAR.S270328 PMC7569248 33116871

