# Supplementary figures and images for: Analysis of PANoptosis-Related LncRNA-miRNA-mRNA Network Reveals LncRNA SNHG7 Involved in Chemo-Resistance in Colon Adenocarcinoma
Source: Front Oncol. 2022 May 12;12:888105. doi: 10.3389/fonc.2022.888105 (PMC9133343; doi:10.3389/fonc.2022.888105)

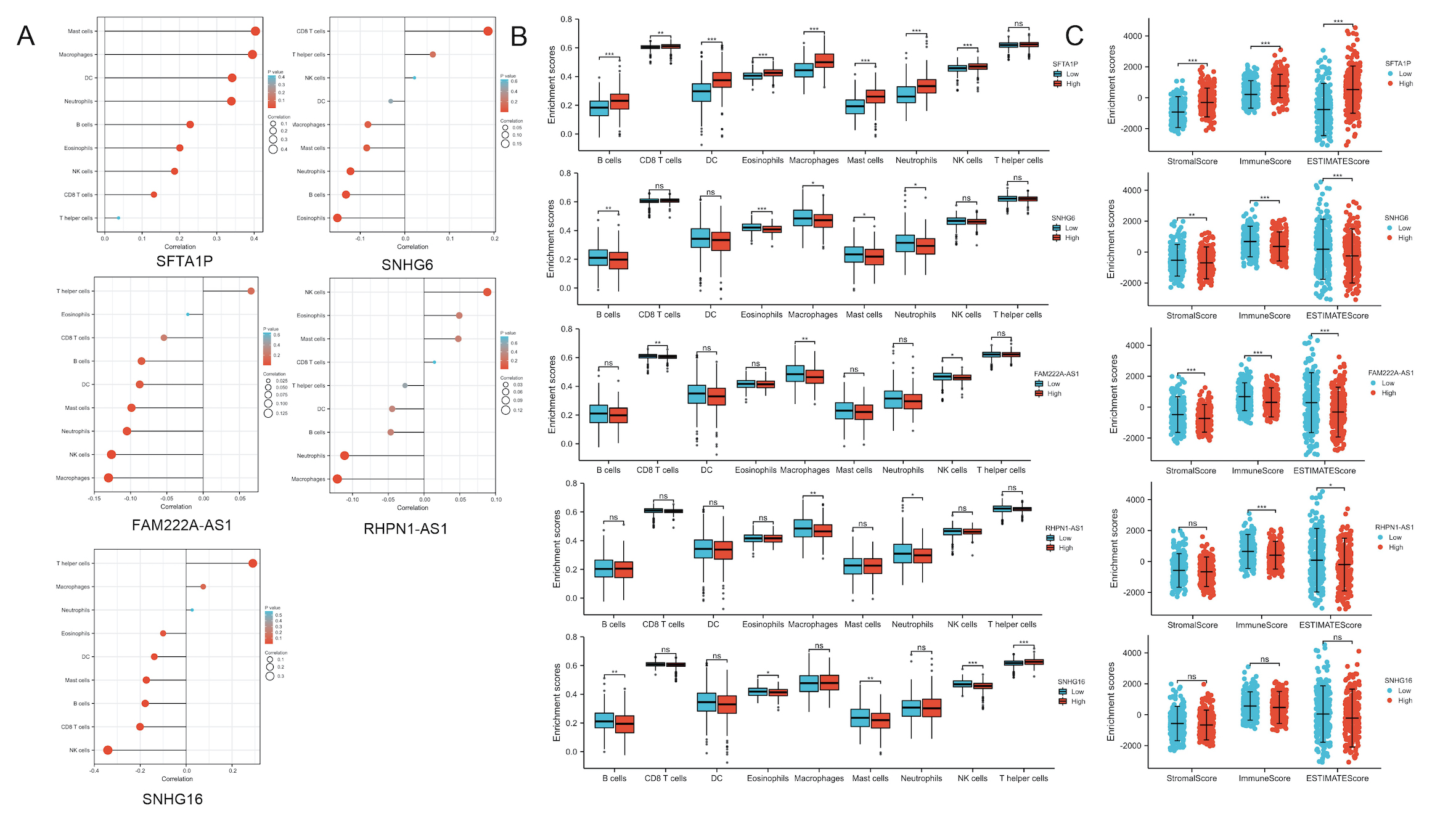

Supplement: Supplementary file 1 [file Image_1.jpeg]

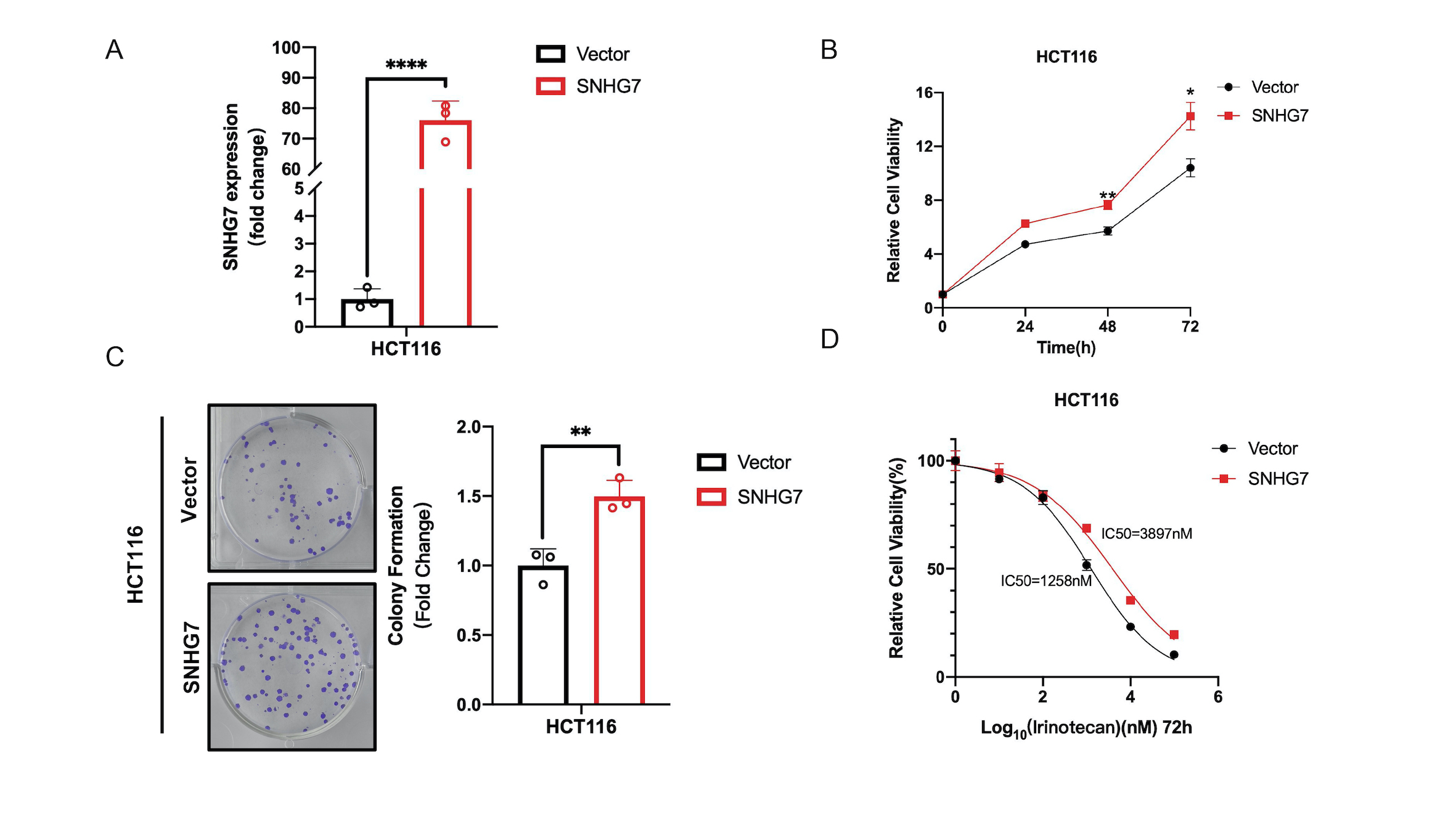

Supplement: Supplementary file 2 [file Image_2.jpeg]
